# Evaluating the Impact of Aevidum on Mental Health Knowledge, Attitudes, and Help-Seeking Behaviors in High School Students: A Mixed-Methods Study

**DOI:** 10.1177/08901171231204473

**Published:** 2023-09-30

**Authors:** Krista L. Pattison, Erik Lehman, Alissa Molinari, Heather Costigan, Francesca Pileggi, Heather Stuckey, Deepa L. Sekhar

**Affiliations:** 1 Department of Pediatrics, 12310 Penn State College of Medicine, Hershey, PA, USA; 2 Department of Public Health Sciences, 12310 Penn State College of Medicine, Hershey, PA, USA; 3 Qualitative and Mixed Methods Core, Penn State College of Medicine, Hershey, PA, USA; 4 Aevidum, Lancaster, PA, USA

**Keywords:** mental health education, peer support, depression

## Abstract

**Purpose:**

To compare Aevidum’s school mental health curriculum vs the curriculum plus Aevidum clubs in a mixed-methods study including pre/post surveys, a randomized clinical trial, and qualitative interviews.

**Design:**

Concurrent mixed-methods: Aim 1) pre-post surveys evaluated curriculum only vs curriculum plus club schools separately regarding changes in knowledge, help-seeking, and school culture; Aim 2) randomized clinical trial compared curriculum only to curriculum plus club schools; Aim 3) qualitative school staff interviews enhanced understanding of school culture changes.

**Setting:**

Curriculum delivered to 9th graders at ten Pennsylvania high schools; 5 schools randomized to start clubs.

**Subjects:**

Students (surveys), staff (interviews).

**Intervention:**

Aevidum curriculum plus/minus club.

**Measures:**

Aim 1, mixed effects linear and logistic regression models for longitudinal data were used to analyze survey items at each time point. Aim 2, the same regression models were used, except models included a fixed-effect for group and group by time interaction effect. Aim 3, interviews were transcribed; a codebook was developed followed by thematic analysis.

**Results:**

Pre-survey 2557 respondents; 49% female, 86% non-Hispanic white. Post-survey 737 (29% response rate). Aim 1, pre-post (Likert responses, larger numbers favorable) demonstrated increased student knowledge to identify depression (4.26 [4.19-4.33] to 4.59 [4.47-4.71], *P* < .001) and help a friend access support (4.30 [4.21-4.38] to 4.56 [4.40-4.71], *P* = .001). Help-seeking increased for phone helplines (1.61 [1.57-1.66] to 1.78 [1.70-1.86], *P* < .001), crisis textlines (1.60 [1.55-1.64] to 1.78 [1.70-1.86], *P* < .001), internet/websites (1.80 [1.75-1.85] to 1.99 [1.90-2.08], *P* < .001), school counselors (*P* = .005) and teachers (.013). Aim 2, no significant differences in knowledge, help-seeking or culture between curriculum only vs curriculum plus club schools. Aim 3, staff (n = 17) interviews supported reduced stigma and increased mental health referrals.

**Conclusions:**

Aevidum’s curriculum improved mental health knowledge and help-seeking; adding the club did not significantly change responses. Staff identified positive school culture impacts. Limitations include the lower post-survey response.

## Purpose

Less than half of United States adolescents experiencing a major depressive episode received treatment in 2020.^1-3^ Explanations include reluctance to discuss mental health with parents and a preference to seek help from peers.^4-6^ Schools have long been identified as an ideal setting to offer mental health intervention and prevention programs, as adolescents, regardless of sex, race/ethnicity, and/or socioeconomic status, spend most of their waking hours in schools.^7-9^

The University of Michigan’s (UM) Peer-2-Peer (P2P) Depression Awareness Program was 1 such school-based initiative that aimed to decrease mental illness and promote well-being.^10^ Students were trained as peer leaders to design and implement a depression awareness campaign. Pre-post assessments demonstrated improved knowledge and attitudes regarding depression, greater confidence to identify and refer at-risk peers, improved help-seeking, and reduced stigma.^10^

Similar to P2P, Aevidum, meaning “I’ve got your back,” is a student-led mental health initiative started in 2010 following a peer suicide in Lancaster, Pennsylvania (PA).^11^ Aevidum has grown from statewide to national reach, and the Aevidum mental health curriculum and club activities are currently used in over 300 schools within PA and surrounding states. Like P2P, Aevidum leverages peer networks in its clubs to expand knowledge about mental health, promote help-seeking, and reduce stigma.^11^ In 2017, Aevidum introduced its school mental health curriculum adopted by many PA schools to meet mandated Act 71 curriculum standards for mental health and suicide prevention.^12^ Despite widespread use, neither the Aevidum curriculum nor club have been formally evaluated.

The study purpose was to understand the effect of the Aevidum curriculum and club on mental health knowledge, help-seeking intentions, and school culture. The study used a mixed-methods design including pre-post surveys, a randomized clinical trial design, and qualitative interviews involving ten Pennsylvania public high schools.

### Aevidum Curriculum and Clubs

The Aevidum Mental Health & Suicide Prevention Curriculum was developed with a team of school counselors, principals, and psychologists and consists of 5 lessons delivered over 3 hours.^13^ The content can be given in 1 three-hour session or broken into smaller segments; videos pair with the lessons. School staff participated in a one-hour educator workshop, and were then asked to deliver the curriculum to 9^th^ graders by February 2022. Due to trimester scheduling, 1 school delivered the curriculum beginning March 2022.

The Aevidum club can be implemented independently of the curriculum. The intent is to foster cultures of caring, kindness, and advocacy.^14^ When starting a club, schools are requested to have approximately ten students and 2 educators attend a 1 day “Talk” training workshop facilitated by students and adult advisors from an active club.

Ten participants allow enough trained students to launch something significant, and schools can send student leaders from different grade levels and social groups. Yet, participation is limited to ten as training often includes multiple schools.

The Talk format has been endorsed by national experts (Substance Abuse & Mental Health Service Organization), and was featured on the top National Prevention Weeks campaigns.^14^ New clubs receive an Aevidum starter kit including banners, posters, and promotional materials, suggested campaign ideas, and wristbands with the National Suicide Prevention Lifeline number.^14^ Schools implementing the club participated in a virtual Talk workshop on October 29^th^, 2021, and were mailed the starter kit. Additionally, schools were asked to carry out 2 club campaigns/activities during the academic year. Schools received a $1k stipend to support club activities, with no restrictions other than activities relate to mental health. Club campaigns have included “Be Kind to Your Mind,” an after-school “hangout” initiative, and “Twosday,” a tutu dress-up day to signify the hourly number (twenty-two) of adolescents who attempt suicide. Additional activities have included spirit weeks and participation in homecoming parades.

## Methods

### Design & Sample

**Figure 1.**
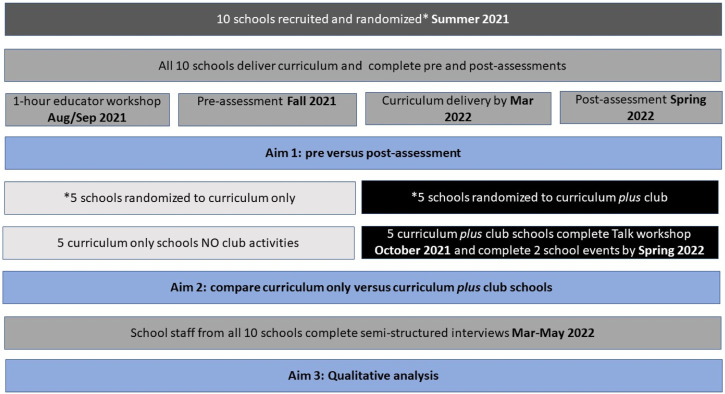
Overview of the components of the mixed-methods study.

**Figure 2.**
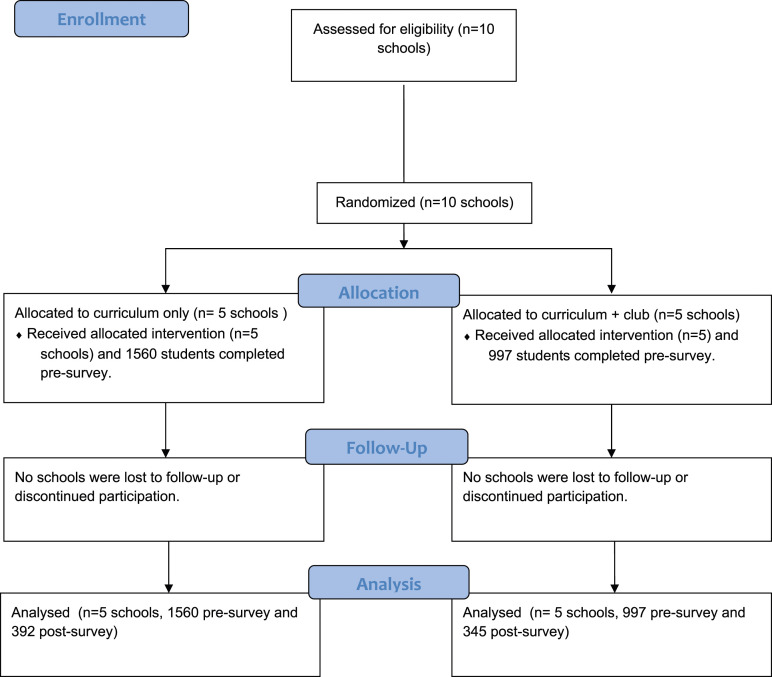
CONSORT flow diagram.

This study took place over the August 2021–June 2022 school year and focused on evaluating Aevidum curriculum and club effectiveness to improve adolescent mental health knowledge, help-seeking intentions, and school culture. Figure 1 overviews the mixed-methods study design. With Aevidum’s Executive Director, ten Pennsylvania schools were recruited via email communication. Priority was given to schools with previous interest in Aevidum and/or a prior relationship with the research team.^15,16^ Before the 2021-2022 academic year, schools were randomly assigned via covariate-constrained randomization to implement the Aevidum curriculum (n = 5) *or* the curriculum and club (n = 5). Curriculum only schools had the opportunity to start clubs the following academic year. This study received an exempt research determination by the Penn State Institutional Review Board. The randomized clinical trial was registered in ClincialTrials.gov (CONSORT diagram Figure 2).

### Measures

Pre-post surveys, used with permission, were those used to evaluate P2P, the UM Depression Center P2P Depression Awareness Assessment.^10,17^ Questions are broadly grouped into mental health and depression knowledge, help-seeking, and school culture changes. While the survey asks no personal information, students were incentivized for pre and post-survey completion. For completing the pre-survey, 4 students per school were chosen to receive a $20 gift card (post-survey $30 gift card). Thus, to be eligible for the raffle, and to ensure post-survey distribution only to those who completed the pre-survey, an email address was required. Opt-out forms were sent to student parents at all participating schools. Prior to Aevidum curriculum implementation, all eligible students received a link to REDCap (Research Electronic Data Capture), a secure, web-based software platform designed to support data capture for research studies.^18,19^ This link was forwarded directly to students by the schools, thus the study team was not provided a list of student opt-outs. Post-surveys were sent via the same secure online system at the conclusion of the school year directly to individual student email addresses supplied following completion of the pre-survey.^18,19^

### Qualitative Component

Survey responses from curriculum only schools were compared to curriculum plus club schools to understand the added benefit of the club on school culture. However, as school culture shifts may be subtle, the student survey data was supplemented with school staff interviews to better understand school culture outcomes from Aevidum. Semi-structured interviews were conducted with up to 5 staff from each school (March 31, 2022 to May 16, 2022). Participants received a $25 gift card. A qualitative interview guide was developed, interviews were conducted via web-based meeting platform (Zoom), audio-recorded, and transcribed for analysis purposes. Transcripts were reviewed and uploaded into NVivo, a qualitative software program, for coding and analysis.

### Analysis

#### Pre- and Post-Surveys

In primary statistical analysis (Aim 1), curriculum only and curriculum plus club schools were analyzed separately, using the same methods. Mixed effects linear and logistic regression models appropriate for longitudinal (repeated measures) data were used to analyze all survey items collected at each time point. The models contained a fixed effect for time (pre vs post) and random effects for school and student. The parameter for time was the primary parameter of interest in the model, as it indicates the change over time with intervention implementation. Parameter estimates and standard errors from the models are reported along with corresponding 95% confidence intervals and *P*-values. The impact on 9^th^ grade, to which the curriculum was delivered, was also considered separately.

In secondary statistical analysis (Aim 2), curriculum only were compared directly to curriculum plus club schools regarding changes in knowledge, help-seeking behavior, and school culture. The same mixed effects regression models were used for each survey item, except the model also included a fixed-effect for group (curriculum only vs curriculum plus club) and a group by time interaction effect. All analyses were performed using SAS statistical software version 9.4 (SAS Institute, Cary, NC).

#### School Staff Semi-Structured Interviews

In Aim 3, the intent was to evaluate school culture change outcomes (eg, stigma) as a result of Aevidum. An inductive, qualitative codebook was developed by the study team after reviewing all transcripts and coming to consensus on common codes and sub-codes. Two independent coders (HC & ML) coded 20% of the data and met to determine the kappa. Substantial agreement was obtained between the 2 coders with a final kappa of .78. One coder (ML) coded the remaining data for subsequent thematic analysis.^20,21^ As in other research this team has completed, the collaborative approach to coding and theming is described in those manuscripts.^22^

## Results

### Pre-Post Surveys (Aim 1)

In total, 2557 students completed the pre-survey; 1560 curriculum only students and 997 curriculum plus club arm students. Mean age was 15.6 years, 49% female, 86% identified as non-Hispanic White, and 30% were 9^th^ graders to whom the curriculum was delivered (Table 1). Post-surveys, sent only to those who completed the pre-survey, were completed by 392 (25%) curriculum only students and 345 (35%) curriculum plus club arm students. Post-survey non-response was significantly higher for males, Black and Hispanic adolescents, and 12^th^ graders.

**Table 1.** Participant Demographics.

Characteristic	Total (N = 2557)	Curriculum only (N = 1560)	Curriculum + club (N = 997)
Student demographics - surveys			
Age, mean ± SD	15.61 ± 1.22	15.55 ± 1.22	15.71 ± 1.22
Missing	1 (<.1%)	1 (<.1%)	0 (.0%)
Gender, N (%)			
Male	1211 (47.4%)	764 (49.0%)	447 (44.8%)
Female	1262 (49.4%)	747 (47.9%)	515 (51.7%)
Other	81 (3.2%)	49 (3.1%)	32 (3.2%)
Missing	3 (.1%)	0 (0.0%)	3 (.3%)
Race/ethnicity, N (%)			
Non-Hispanic white	2191 (85.7%)	1305 (83.7%)	886 (88.9%)
Non-Hispanic Black	48 (1.9%)	35 (2.2%)	13 (1.3%)
Hispanic	199 (7.8%)	148 (9.5%)	51 (5.1%)
Non-Hispanic Asian	38 (1.5%)	17 (1.1%)	21 (2.1%)
Non-Hispanic other	72 (2.8%)	47 (3.0%)	25 (2.5%)
Missing	9 (.4%)	8 (.5%)	1 (.1%)
Grade, N (%)			
9^th^	758 (29.6%)	506 (32.4%)	252 (25.3%)
10^th^	640 (25.0%)	398 (25.5%)	242 (24.3%)
11^th^	617 (24.1%)	369 (23.7%)	248 (24.9%)
12^th^	532 (20.8%)	279 (17.9%)	253 (25.4%)
Missing	10 (.4%)	8 (.5%)	2 (.2%)

Pre-to post-survey comparisons within study arms for all grades and 9^th^ grade alone are shown in Tables 2 and 3. Overall, curriculum only students reported improvements in mental health knowledge with significant increases in confidence to identify someone showing common signs of depression (7-point Likert scale with larger numbers more favorable, mean 4.26 [4.19-4.33] to 4.59 [4.47-4.71], *P* < .001) and ability to help a friend access school and/or community mental health supports (4.30 [4.21-4.38] to 4.56 [4.40-4.71], *P* = .001). In particular, 9^th^ graders indicated higher levels of confidence for both items (post-survey 4.63 [4.46-4.81] and 4.77 [4.55-4.98] respectively). Students demonstrated greater understanding that depression is not a sign of personal weakness (correct question response 77% to 86%, *P* < .001). Further, mental health literacy gains were significant in more students being able to identify depression symptoms, specifically, difficulty concentrating, feeling angry, frequent unexplained aches and pains, feeling tired or less energetic, and/or eating more than usual.

**Table 2.** Comparisons of Pre-to Post-survey Responses for Knowledge and Help-seeking – Curriculum Only Schools.

	All grades (N = 1560)			9^th^ grade only (N = 506)		
Item	Pre (N = 1560)	Post (N = 392)	*P*-value	Pre (N = 506)	Post (N = 190)	*P*-value
Knowledge questions						
How confident are you in your ability to identify someone who is showing the common signs of depression?	4.26 (4.19, 4.33)	4.59 (4.47, 4.71)	<.001	4.13 (4.01, 4.24)	4.63 (4.46, 4.81)	<.001
Responses: 1 (Not at all confident), 2, 3, 4 (Moderately confident), 5, 6, 7 (Extremely confident)^a^						
How confident are you in your ability to help a friend access mental health support services in your school or in the community?	4.30 (4.21, 4.38)	4.56 (4.40, 4.71)	.001	4.40 (4.26, 4.54)	4.77 (4.55, 4.98)	.002
Responses: 1 (Not at all confident), 2, 3, 4 (Moderately confident), 5, 6, 7 (Extremely confident)^a^						
If your friend tells you that he/she is thinking about suicide and asks you to keep it a secret because no 1 else knows, what do you do?	1353 (87.2%)	350 (89.7%)	.108	434 (86.3%)	172 (91.0%)	.063
Responses: tell someone (correct), keep it a secret^b^						
Depression runs in some families	1288 (82.8%)	344 (87.8%)	.016	404 (80.0%)	161 (84.74%)	.148
Responses: true (correct), false^b^						
Depression can be controlled through willpower	956 (61.7%)	277 (70.7%)	.007	299 (59.3%)	128 (67.4%)	.148
Responses: true, false (correct)^b^						
Depression is a treatable health condition	1262 (81.4%)	318 (81.3%)	.335	392 (78.1%)	157 (83.1%)	.033
Responses: true (correct), false^b^						
The abuse of alcohol and drugs can be a sign of depression	1453 (93.6%)	377 (96.2%)	.057	463 (92.1%)	185 (97.4%)	.017
Responses: true (correct), false^b^						
Depression is a sign of personal weakness	1191 (76.8%)	335 (85.5%)	<.001	368 (73.2%)	158 (83.2%)	.013
Responses: true, false (correct)^b^						
Which of the items below could be symptoms of depression if they continue for more than 2 weeks? (check all that apply)						
Difficulty concentrating or making decisions^b^	1283 (82.2%)	343 (87.5%)	.020	413 (81.6%)	170 (89.5%)	.011
Feeling angry^b^	1173 (75.2%)	318 (81.1%)	.008	361 (71.3%)	159 (83.7%)	<.001
Changes in sleep patterns^b^	1413 (90.6%)	366 (93.4%)	.097	455 (89.9%)	176 (92.6%)	.306
Frequent unexplained aches and pains^b^	677 (43.4%)	230 (58.7%)	<.001	194 (38.3%)	111 (58.4%)	<.001
Feeling tired or less energetic^b^	1447 (92.8%)	377 (96.2%)	.017	464 (91.7%)	182 (95.8%)	.068
Eating more than usual^b^	1058 (67.8%)	315 (80.4%)	<.001	333 (65.8%)	152 (80.0%)	<.001
Feeling irritable or restless^b^	1335 (85.6%)	350 (89.3%)	.059	426 (84.2%)	164 (86.3%)	.550
Help seeking questions						
If you had symptoms of depression that lasted for more than 2 weeks, would you ask for help?	832 (53.7%)	217 (55.8%)	.336	271 (53.9%)	109 (57.7%)	.116
Responses: yes (correct), no^b^						
If you thought that you had depression, how likely is it that you would seek help from the following people or places?Response scale: 1 (Not at all likely), 2 (Not too likely), 3 (Somewhat likely), 4 (Very likely)						
Friend^a^	3.07 (3.03, 3.11)	3.12 (3.04, 3.20)	.252	3.02 (2.94, 3.10)	3.09 (2.97, 3.21)	.249
Parent or guardian^a^	2.70 (2.65, 2.76)	2.71 (2.62, 2.79)	.928	2.71 (2.61, 2.81)	2.74 (2.61, 2.87)	.566
School counselor^a^	2.01 (1.96, 2.06)	2.14 (2.05, 2.23)	.005	2.01 (1.92, 2.10)	2.17 (2.04, 2.30)	.015
Teacher^a^	1.95 (1.90, 2.00)	2.06 (1.97, 2.14)	.013	1.87 (1.79, 1.95)	2.01 (1.89, 2.14)	.026
Mental health professional (ie, psychologist, psychiatrist, social worker)^a^	2.64 (2.58, 2.69)	2.68 (2.59, 2.78)	.353	2.64 (2.55, 2.74)	2.68 (2.54, 2.83)	.598
Doctor^a^	2.39 (2.34, 2.44)	2.39 (2.30, 2.48)	.992	2.44 (2.35, 2.53)	2.45 (2.31, 2.59)	.819
Internet/website^a^	1.80 (1.75, 1.85)	1.99 (1.90, 2.08)	<.001	1.74 (1.65, 1.83)	1.98 (1.84, 2.11)	<.001
Clergy, priest, rabbi, or other religious person^a^	1.65 (1.60, 1.69)	1.59 (1.51, 1.67)	.125	1.72 (1.64, 1.80)	1.68 (1.56, 1.80)	.564
Phone helpline^a^	1.61 (1.57, 1.66)	1.78 (1.70, 1.86)	<.001	1.66 (1.58, 1.74)	1.85 (1.72, 1.97)	.006
Crisis textline^a^	1.60 (1.55, 1.64)	1.78 (1.70, 1.86)	<.001	1.65 (1.57, 1.72)	1.83 (1.71, 1.96)	.006
Other relative (ie, sister, brother, aunt, uncle)^a^	2.64 (2.59, 2.69)	2.61 (2.51, 2.70)	.509	2.69 (2.60, 2.79)	2.68 (2.54, 2.82)	.894
Boyfriend or girlfriend^a^	2.99 (2.94, 3.04)	2.97 (2.88, 3.07)	.746	2.91 (2.82, 3.00)	2.96 (2.82, 3.10)	.505
Coach^a^	1.97 (1.92, 2.03)	1.97 (1.89, 2.06)	.956	2.08 (1.99, 2.18)	2.15 (2.02, 2.28)	.314
Do you know how to get help in your school?	2.31 (2.27, 2.34)	2.42 (2.35, 2.48)	<.001	2.31 (2.25, 2.36)	2.48 (2.39, 2.57)	<.001
Responses: 1 (Not really), 2 (Sort of), 3 (Definitely)^a^						
If you thought you had mental health issues, is there a trusted adult you would feel comfortable going to? Responses: yes (% shown), no^b^	1255 (81.7%)	331 (85.8%)	.066	406 (81.4%)	161 (87.0%)	.086

^a^Mean (95% CI) and *P*-values from a mixed effects linear regression model.

^b^N (%); *P*-values from a mixed effects logistic regression model.

**Table 3.** Comparisons of Pre-to Post-survey Responses for Culture/climate – Curriculum + Club Schools.

	All grades (N = 997)			9^th^ grade only (N = 252)		
Item	Pre (N = 997)	Post (N = 345)	*P*-value	Pre (N = 252)	Post (N = 87)	*P*-value
Culture/climate						
On a scale from 1 to 7, if you were seen going into the office of your school social worker or school psychologist, how would you feel?	3.51 (3.39, 3.62)	3.30 (3.12, 3.48)	.028	3.69 (3.46, 3.93)	3.41 (3.05, 3.77)	.109
Responses: 1 (Not at all embarrassed), 2, 3, 4 (Moderately embarrassed), 5, 6, 7 (Extremely embarrassed)^a^						
Imagine that you recently heard about a new student at your school who has depression. To what extent do you agree or disagree with the following statements?Response: 1 (Strongly disagree), 2 (Disagree), 3 (Neither agree nor disagree), 4 (Agree), 5 (Strongly agree)						
The new student is more dangerous than other students^a^	1.98 (1.92, 2.04)	2.13 (2.04, 2.23)	.003	2.04 (1.92, 2.16)	2.37 (2.18, 2.56)	<.001
The student is to blame for his or her condition^a^	1.66 (1.60, 1.71)	1.72 (1.64, 1.81)	.147	1.80 (1.68, 1.91)	2.02 (1.84, 2.19)	.009
I would have sympathy for the new student^a^	3.78 (3.73, 3.84)	3.71 (3.62, 3.79)	.077	3.72 (3.61, 3.83)	3.62 (3.44, 3.79)	.245
The new student makes me feel scared^a^	1.74 (1.69, 1.79)	1.81 (1.73, 1.90)	.116	1.81 (1.70, 1.92)	2.04 (1.87, 2.21)	.009
The new student makes me feel uncomfortable^a^	1.87 (1.82, 1.93)	1.89 (1.79, 1.98)	.821	1.96 (1.84, 2.08)	2.15 (1.96, 2.33)	.047
I would help the new student even if I did not know him or her well^a^	3.78 (3.72, 3.83)	3.56 (3.48, 3.65)	<.001	3.77 (3.65, 3.88)	3.43 (3.27, 3.60)	<.001
I would try to stay away from the new student^a^	1.97 (1.92, 2.03)	2.09 (2.00, 2.17)	.012	2.03 (1.92, 2.14)	2.24 (2.06, 2.41)	.029
The new student would be made fun of at my school^a^	2.77 (2.71, 2.84)	2.96 (2.87, 3.06)	<.001	2.73 (2.61, 2.85)	3.19 (2.99, 3.38)	<.001
The new student would be ignored at my school^a^	2.85 (2.79, 2.91)	3.03 (2.93, 3.12)	<.001	2.67 (2.56, 2.78)	3.05 (2.86, 3.23)	<.001
I think other students in my school would try to help the new student^a^	3.28 (3.22, 3.34)	3.12 (3.02, 3.22)	.002	3.45 (3.33, 3.56)	3.19 (3.01, 3.37)	.008
How comfortable are you talking about mental health issues with other students at your school?	3.51 (3.40, 3.62)	3.66 (3.48, 3.84)	.115	3.29 (3.08, 3.51)	3.23 (2.90, 3.56)	.706
Response scale: 1 (Not at all comfortable), 2, 3, 4 (Moderately comfortable), 5, 6, 7 (Extremely comfortable)^a^						
What mental health issues do you think are most concerning to other students at your school? Check all that apply						
Depression^b^	813 (81.5%)	282 (81.7%)	.978	199 (79.0%)	65 (74.7%)	.283
Anxiety^b^	762 (76.4%)	267 (77.4%)	.824	184 (73.0%)	67 (77.0%)	.471
Thoughts of suicide^b^	712 (71.4%)	256 (74.2%)	.443	178 (70.6%)	59 (67.8%)	.579
I do not think there are any mental health issues that are concerning for students^b^	79 (7.9%)	34 (9.9%)	.257	20 (7.9%)	9 (10.3%)	.494
Other^b^	61 (6.1%)	84 (24.4%)	<.001	17 (6.8%)	21 (24.1%)	<.001
How much do your teachers know about addressing student mental health needs?	1.85 (1.81, 1.89)	1.86 (1.80, 1.93)	.679	1.96 (1.88, 2.04)	2.04 (1.90, 2.17)	.299
Responses: 1 (A little), 2 (some), 3 (A lot)^a^						
How much do your school counselors know about addressing student mental health needs?	2.31 (2.27, 2.36)	2.30 (2.22, 2.37)	.643	2.43 (2.34, 2.52)	2.42 (2.28, 2.56)	.934
Responses: 1 (A little), 2 (some), 3 (A lot)^a^						
How would you like your school to show that they care about student mental health? Check all that apply:						
Mental health information sheets at school^b^	429 (43.0%)	150 (43.5%)	.966	99 (39.3%)	24 (27.6%)	.023
Class activities about mental health^b^	516 (51.8%)	168 (48.7%)	.275	118 (46.8%)	37 (42.5%)	.570
Mental health information on school website^b^	593 (59.5%)	201 (58.3%)	.288	145 (57.5%)	42 (48.3%)	.136
Other^b^	125 (12.5%)	113 (32.8%)	<.001	36 (14.3%)	35 (40.2%)	<.001
On average, how often do your teachers speak to you about your emotions and feelings?	3.33 (3.28, 3.38)	3.34 (3.25, 3.42)	.851	3.08 (2.95, 3.20)	3.26 (3.06, 3.46)	.093
Responses: 1 (daily), 2 (weekly), 3 (monthly), 4 (never)^a^						
Would you like your teachers to speak to you about your emotions and feelings?	2.57 (2.53, 2.60)	2.52 (2.46, 2.58)	.197	2.62 (2.54, 2.69)	2.52 (2.39, 2.65)	.168
Responses 1 (yes), 2 (maybe), 3 (no)^a^						

^a^Mean (95% CI) and *P*-values from a mixed effects linear regression model.

^b^N (%); *P*-values from a mixed effects logistic regression model.

Improvements in help-seeking were less pronounced, though students were significantly more likely to report intention to seek help from phone helplines (4-point Likert scale with larger numbers more favorable, 1.61 [1.57-1.66] to 1.78 [1.70-1.86], *P* < .001), crisis textlines (1.60 [1.55-1.64] to 1.78 [1.70-1.86], *P* < .001), internet/websites (1.80 [1.75-1.85] to 1.99 [1.90-2.08], *P* < .001), and school counselors (*P* = .005) and teachers (.013).

Curriculum plus club schools were analyzed for changes to school culture and climate with mixed results. Students reported decreased embarrassment if seen entering the counselor’s office on the post-test (7-point Likert scale with lower numbers more favorable 3.51 [3.39-3.62] to 3.30 [3.12-3.48], *P* = .028). Yet, these same students responded that a new student with depression would be viewed as dangerous (5-point Likert scale with lower numbers more favorable 1.98 [1.92-2.04] to 2.13 [2.04-2.23], *P* = .003). Students indicated they would be disinclined to help this student and more likely to stay away. Students reported the new student would be likely to be made fun of (2.77 [2.71-2.84] to 2.96 [2.87-3.06], *P* < .001) or ignored (2.85 [2.79-2.91] to 3.03 [2.93-3.12], <.001). These negative feelings towards a peer with depression were more pronounced for 9^th^ graders. Finally, on the post-survey more students indicated “other” mental health problems were an issue and they would like the school to show they care about student mental health in “other” ways. Free text responses were not available for additional detail.

### Curriculum Only vs Curriculum Plus Club (Aim 2)

Comparison of responses from curriculum only to curriculum plus club schools yielded essentially no differences in pre-post test responses between groups as relates to knowledge, except those in the curriculum only arm had significantly greater odds of recognizing that feeling tired or less energetic could be a symptom of depression (OR [95% CI] for curriculum 1.88 [1.12-3.15] vs curriculum plus club schools .82 [.50-1.36], *P* = .025). Regarding help-seeking, students in the curriculum plus club arm were more likely to seek help from clergy (difference of means [95% CI] for curriculum -.06 [-.14-.02] vs curriculum plus club schools .07 [-.02-.15], *P* = .03). Students in the curriculum only arm demonstrated more of a positive response to a new peer with depression than those in the curriculum plus club arm.

### School Staff Interviews (Aim 3)

Interviews were conducted with staff from 7/10 schools. Roles included administrator (n = 1), teacher (n = 11), counselor (n = 2), school nurse (n = 2), and social worker (n = 1). School staff were non-Hispanic white, mostly female (n = 13), aged 50-59 (n = 8), and held a Master’s degree (n = 13). Length in their current roles ranged from 1 to over 20 years.

Three major themes are described below and in Table 4 where they are mapped against survey findings.

**Table 4.** Comparisons of Change From Pre-to Post-survey Responses Between Study Arms (all Grades).

Item	Curriculum only (N = 1560)	Curriculum + club (N = 997)	*P*-value	Qualitative results
Knowledge questions				
How confident are you in your ability to identify someone who is showing the common signs of depression?	.33 (.21, .45)	.29 (.16, .42)	.642	
Responses: 1 (Not at all confident), 2, 3, 4 (Moderately confident), 5, 6, 7 (Extremely confident)^a^				
How confident are you in your ability to help a friend access mental health support services in your school or in the community?	.26 (.11, .42)	.24 (.07, .41)	.864	
Responses: 1 (Not at all confident), 2, 3, 4 (Moderately confident), 5, 6, 7 (Extremely confident)^a^				
If your friend tells you that he/she is thinking about suicide and asks you to keep it a secret because no 1 else knows, what do you do?	1.32 (.94, 1.84)	1.70 (1.15, 2.50)	.336	
Responses: tell someone (correct), keep it a secret^b^				
Depression runs in some families	1.41 (1.06, 1.86)	1.34 (.99, 1.80)	.806	
Responses: true (correct), false^b^				
Depression can be controlled through willpower	1.31 (1.07, 1.60)	1.37 (1.09, 1.71)	.781	
Responses: true, false (correct)^b^				
Depression is a treatable health condition Responses: true (correct), false^b^	1.12 (.86, 1.46)	1.21 (.89, 1.64)	.720	
The abuse of alcohol and drugs can be a sign of depression	1.71 (.98, 2.97)	1.54 (.91, 2.59)	.784	
Responses: true (correct), false^b^				
Depression is a sign of personal weakness Responses: true, false (correct)^b^	1.49 (1.19, 1.85)	1.37 (1.03, 1.82)	.662	
Which of the items below could be symptoms of depression if they continue for more than 2 weeks? (check all that apply)				
Difficulty concentrating or making decisions^b^	1.41 (1.05, 1.88)	1.75 (1.24, 2.47)	.340	
Feeling angry^b^	1.39 (1.09, 1.77)	1.71 (1.30, 2.25)	.265	
Changes in sleep patterns^b^	1.38 (.92, 2.05)	1.96 (1.23, 3.11)	.259	
Frequent unexplained aches and pains^b^	1.91 (1.57, 2.34)	1.71 (1.39, 2.10)	.450	
Feeling tired or less energetic^b^	1.88 (1.12, 3.15)	.82 (.50, 1.36)	.025	
Eating more than usual^b^	1.92 (1.52, 2.43)	1.90 (1.45, 2.47)	.937	
Feeling irritable or restless^b^	1.34 (.99, 1.81)	1.61 (1.12, 2.32)	.447	
Help seeking questions				Help seeking qualitative school staff interviews
If you had symptoms of depression that lasted for more than 2 weeks, would you ask for help?	1.10 (.91, 1.32)	.86 (.71, 1.05)	.086	Theme 1: Overall, there seemed to be an increase in mental health referrals at schools, due to Aevidum as well as increasing acceptance of speaking to counselors
Responses: yes (correct), no (incorrect)^b^				
If you thought that you had depression, how likely is it that you would seek help from the following people or places? Response scale: 1 (Not at all likely), 2 (Not too likely), 3 (Somewhat likely), 4 (Very likely)				The prioritization of mental health was discussed, with a perceived increase in mental health referrals and connecting students with needed services:
Friend^a^	.05 (-.03, .13)	.03 (-.06, .12)	.800	
Parent or guardian^a^	.0 (-.08, .09)	.05 (-.04, .15)	.451	
School counselor^a^	.13 (.03, .22)	.06 (-.04, .16)	.335	*I've had more students this year say, “Well, I wasn't going to come in, but my friend talked to me about it and really recommend I come in,” or they come in together with their moral support person. And that's been happening more this year, I think, than in previous years. So I would say that Aevidum is having something to do with that, because I think the whole willing to talk about their mental health issues is increased partially due to Aevidum. And then I think being willing to come in and address those issues and address their concerns or their concerns about their friends also is related to that*.
Teacher^a^	.11 (.02, .20)	.15 (.05, .24)	.554	
Mental health professional (ie, psychologist, psychiatrist, social worker)^a^	.05 (-.05, .14)	.09 (-.01, .19)	.541	
Doctor^a^	.0 (-.09, .09)	-.05 (-.15, .05)	.454	*But I know from just anecdotal looks in the classroom, I do notice more kids seeking help, more kids that have been going out for even * *long-* *term* * placement to get the help that they need more so than has been in the past.*
Internet or website^a^	.19 (.09, .28)	.21 (.11, .31)	.749	
Clergy, priest, rabbi, or other religious person^a^	-.06 (-.14, .02)	.07 (-.02, .15)	.030	
Phone helpline^a^	.17 (.08, .25)	.11 (.02, .20)	.334	Some schools were not as sure about Aevidum’s impact and discussed mental health awareness more generally:
Crisis textline^a^	.19 (.10, .27)	.11 (.02, .20)	.243	
Other relative (ie, sister, brother, aunt, uncle)^a^	-.03 (-.13, .06)	.02 (-.08, .12)	.492	
Boyfriend or girlfriend^a^	-.02 (-.11, .08)	.02 (-.08, .12)	.610	*I don't know for certain, but I would assume that there was an increase in referrals, due to the circumstances that we dealt with last year and this year with the pandemic, with virtual learning. All that time at home and quarantining and isolation I think caused some mental health issues kind of emphasized those maybe a little more than normally.*
Coach^a^	-.01 (-.09, .08)	.12 (.02, .21)	.061	
Do you know how to get help in your school?	.11 (.05, .17)	.05 (-.02, .12)	.199	
Responses: 1 (Not really), 2 (Sort of), 3 (Definitely)^a^				*We, we have referrals through all the grades. I think all of them are up in referrals, not just specifically the ninth graders. So it's hard to determine if it was attributed to the [Aevidum] curriculum being introduced in them having that awareness or if it was just, “We have these counselors now available, let's do something with it.”*
If you thought you had mental health issues, is there a trusted adult you would feel comfortable going to?	1.28 (.99, 1.67)	1.14 (.86, 1.50)	.526	
Responses: yes, no^b^				
Culture/climate				Culture/climate school staff qualitative interviews
On a scale from 1 to 7, if you were seen going into the office of your school social worker or school psychologist, how would you feel?	-.16 (-.33, .02)	-.21 (-.39, -.02)	.718	Theme 2: Schools reported a noticeable difference of mental health awareness in staff and students, through openness and conversation, visual aids, and an overall positive school climate.
Responses: 1 (Not at all embarrassed), 2, 3, 4 (Moderately embarrassed), 5, 6, 7 (Extremely embarrassed)^a^				
Imagine that you recently heard about a new student at your school who has depression. To what extent do you agree or disagree with the following statements?Response: 1 (strongly disagree), 2 (disagree), 3 (neither agree nor disagree), 4 (agree), 5 (strongly agree)				There was a perceived difference in awareness of resources and communication about mental health & wellness within the participating schools.
The new student is more dangerous than other students^a^	-.10 (-.19, -.01)	.15 (.05, .25)	<.001	Some schools discussed a more general awareness and increase in communication:
The student is to blame for his or her condition^a^	-.11 (-.19, -.03)	.07 (-.02, .15)	.003	
I would have sympathy for the new student^a^	-.02 (-.10, .06)	-.08 (-.16, .01)	.310	*For me, I would say just awareness and just knowing that they can have that conversation and they can say something and being able to identify supports and knowing that I'm available. And there's someone here that can have that conversation with them in a safe place, so creating a dialogue, I think and that openness to have those conversations.*
The new student makes me feel scared^a^	-.02 (-.10, .06)	.07 (-.01, .16)	.114	
The new student makes me feel uncomfortable^a^	.03 (-.05, .12)	.01 (-.08, .10)	.738	
I would help the new student even if I did not know him or her well^a^	-.09 (-.17, -.01)	-.21 (-.30, -.13)	.040	*And I do think that since the pandemic and since we’ve implemented advisory and added Aevidum, I do think that we’re seeing more kids willing and interested and able, or maybe just reaching their breaking points that are able to open up and talk about it than we have before.*
I would try to stay away from the new student^a^	-.06 (-.14, .03)	.12 (.03, .21)	.006	
The new student would be made fun of at my school^a^	.17 (.08, .26)	.19 (.09, .29)	.798	
The new student would be ignored at my school^a^	.08 (-.01, .17)	.17 (.07, .27)	.197	Other comments targeted specifics, such as increase in visual awareness which aided in a more positive school climate:
I think other students in my school would try to help the new student^a^	-.13 (-.22, -.04)	-.16 (-.26, -.06)	.623	
How comfortable are you talking about mental health issues with other students at your school?	.36 (.19, .53)	.15 (-.04, .33)	.090	
Response scale: 1 (Not at all comfortable), 2, 3, 4 (Moderately comfortable), 5, 6, 7 (Extremely comfortable)^a^				
What mental health issues do you think are most concerning to other students at your school? Check all that apply				*We participated in the homecoming parade. The kids were very enthusiastic. … We bought Aevidum clothing, so a lot of them wore that. We wore black and yellow. We handed out cards that had suicide prevention hotline on one side, and our Aevidum logo on the other. That got them so pumped up that they couldn't wait to do the next parade, … We handed out candy canes that we had taped a message to it that had the Aevidum logo on one side, and then the saying, “If you needed a sign to not kill yourself, this is it.”*
Depression^b^	.98 (.76, 1.28)	1.0 (.76, 1.32)	.944	
Anxiety^b^	1.22 (.94, 1.57)	1.03 (.81, 1.33)	.369	
Thoughts of suicide^b^	1.18 (.95, 1.46)	1.10 (.85, 1.41)	.676	*...I think Aevidum certainly has helped because it's putting out there everywhere, “It's okay. It's okay to talk about it. There are people to help you.” We've always done that but we didn't have it plastered all over the walls of the hallway, particularly the ninth grade hallway. They'll put stuff up throughout the building, things that are like, “You're okay. If you need help, get help. This is how to do that.” And things like that. So I think that definitely has helped increase the number of students who have come in for help or who have asked for help.*
I do not think there are any mental health issues that are concerning for students^b^	1.01 (.67, 1.53)	1.28 (.84, 1.94)	.432	
Other^b^	4.24 (3.16, 5.68)	4.87 (3.51, 6.77)	.533	
How much do your teachers know about addressing student mental health needs?	.0 (-.06, .07)	.02 (-.05, .09)	.769	There was also discussion of how Aevidum may have impacted the stigma associated with mental health issues:
Responses: 1 (A little), 2 (some), 3 (A lot)^a^				
How much do your school counselors know about addressing student mental health needs?	.04 (-.03, .11)	-.02 (-.09, .06)	.315	
Responses: 1 (A little), 2 (some), 3 (A lot)^a^				
How would you like your school to show that they care about student mental health? Check all that apply:				
Mental health information sheets at school^b^	.86 (.70, 1.06)	1.0 (.79, 1.26)	.345	*Aevidum really helped keep that conversation going and did take away some of the stigma of it. We talked about that often in the classroom when we were doing the program, like there's no stigma to this anymore. Like don't mock somebody because they're talking about mental health*.
Class activities about mental health^b^	.86 (.71, 1.05)	.88 (.70, 1.10)	.899	
Mental health information on school website^b^	.97 (.78, 1.20)	.89 (.71, 1.12)	.612	
Other^b^	3.84 (3.01, 4.89)	3.36 (2.55, 4.42)	.476	*It’s not taboo to talk about. Everybody experiences struggles and mental health struggles, that's a huge 1 and it was made worse by COVID and I think we are more accepting of them now, now that people were out in the open with it more so I guess, and I think Aevidum allowed our school to have a spot for those kids.*
On average, how often do your teachers speak to you about your emotions and feelings? (daily) Responses: 1 (daily), 2 (weekly), 3 (monthly), 4 (never)^a^	-.08 (-.16, .0)	.01 (-.08, .10)	.141	
Would you like your teachers to speak to you about your emotions and feelings?	.0 (-.07, .06)	-.04 (-.11, .02)	.392	
Responses 1 (yes), 2 (maybe), 3 (no)^a^				

^a^Difference of Means (95% CI) and *P*-values from a mixed effects linear regression model.

^b^Odds Ratio (95% CI) and *P*-values from a mixed effects logistic regression model.

Theme 1 Overall, there seemed to be an increase in mental health referrals at schools, due to Aevidum as well as increasing acceptance of speaking to counselors.

While students did not demonstrate an increase in help-seeking behavior based on survey responses, school staff subjectively reported an increase in student mental health referrals suggesting an impact that was not captured by the survey alone.

Theme 2 Schools reported a noticeable difference of mental health awareness in staff and students, through openness and conversation, visual aids, and an overall positive school climate.

While students did not report many significant changes in school culture/climate from Aevidum, interviews suggest staff did observe a positive change in the school mental health dialogue.

Theme 3 Schools described multiple barriers that get in the way of educating students on mental health, including community hesitancy and a focus on academics in school.

This barriers-related theme was not part of the student survey, but emerged in the staff interviews. Staff described barriers that inhibited mental health awareness efforts. Pushback from “old school” community members that schools should not be dealing with these issues was described:

*And so the barrier that we face a lot of the times is our community thinks and still thinks that schools should just focus on education; education and academics. You shouldn’t be meddling in these kids personal lives. You shouldn’t be dealing with their social, emotional, you shouldn’t be dealing with their mental health.*

*One* *of the biggest barriers may be just traditional values and viewpoints, meaning more of an old school perspective, that a lot of our students get from their homes. So if you’re somehow able to shift the family or parents’ perspective, that’s a good, but that’s awful challenging. So I would think just maybe that’s the biggest barrier is just people stuck in that older school mentality of not speaking up about mental health, or trying to tough things out or not recognizing* *that.*

Time was another implementation barrier, as time for mental health takes away from educational aspects:

*And then the teachers are being told, “You have to do bio remediation, you have to do this. You have to get them ready for keystones.” Testing is part of life and it’s part of like what we do in the high school, but it just seems like more time is allotted to all the academic and the testing than there would be to any mental* *health/behavior* *change with Positive Behavioral Interventions and Supports (PBIS), so I think we get shortchanged…[for]…mental health.*

*I think just because it’s so new, the standardized testing, I think that’s crazy. The kids are going through so much and we’re hitting them with tests. And I think that some kids, academics isn’t their thing. They’re not worried about that if they’re having mental health struggles and I don't think it’s fair to give everybody the same... So I think standardized testing and a standardized test driven society is something that’s... It’s unfair and I think that could be a barrier.*

## Discussion

This mixed-methods evaluation of Aevidum’s mental health curriculum and club was undertaken to evaluate benefits for knowledge, help-seeking, and school culture. Ninth graders who received the curriculum and other students in the school demonstrated both improved mental health knowledge and help-seeking intentions (Aim 1).

Addition of the club did not greatly impact knowledge or help-seeking. Students at curriculum plus club schools reported decreased embarrassment if seen going to the counselor’s office, but overall support for a new peer with depression was poor (Aim 2). Yet, school staff qualitative data suggests visible improvements in school culture not well-captured on the student survey (Aim 3). The findings support mental health education in schools, confirm the value of the Aevidum curriculum to improve high school student knowledge and help-seeking behavior, and point to benefits from a school staff perspective in a positive school culture shift.

## Comparison to Prior Research

Findings are similar to those from to P2P.^10^ P2P students reported higher baseline knowledge and greater baseline and post-survey willingness to seek help than students in our study.^10^ Yet, both programs demonstrated significant change in confidence to identify someone with signs of depression and help a friend access mental health supports. Also similar were improvements in knowledge regarding signs of depression.^10^ Like Aevidum, from pre to post-survey, those exposed to P2P reported help-seeking intention increased with teachers, websites, and helplines, but also for mental health professionals, doctors, and clergy members. In contrast, students exposed to P2P were more likely to positively support a new peer with depression and reported increased comfort talking about mental health issues.^10^ Differences may be due to several factors. First, the P2P analysis discarded all pre-survey results without a corresponding post-survey.^10^ In contrast, we retained all pre-survey results based on the concern that removing those who failed to complete the post-survey might result in a less representative subset of students. P2P student trainee activities required review and approval by the research team.^10^ In contrast, following the Talk training, Aevidum club activities were managed by individual schools. Finally, P2P evaluation was conducted during the 2015-2016 school year,^10^ and our study was conducted during the 2021-2022 school year, complicated by COVID-19. It is unclear if student club participation was limited and how this impacted findings. Several schools were still working through test to stay strategies to maintain in-person learning.^23,24^

Similar results are reported from other school-based depression curricula. The Adolescent Depression Awareness Program (ADAP) was a three-hour curriculum delivered to 710 high school students from 6 Oklahoma schools. ADAP demonstrated improved knowledge and help-seeking, but only among those exposed directly to the programming.^25^ The Integrated Science Education Outreach (InSciEdOut) program was a school-based mental health intervention to reduce stigma delivered to at-risk 7^th^ and 8^th^ grade students in 2015-2016. Participants demonstrated improvements in knowledge and help-seeking, but stigma, which was low at baseline, was unchanged.^26^

The challenge in assessing stigma is that it relates to school culture, which may be difficult to capture in a survey. This was the rationale for inclusion of a qualitative component in this evaluation. School culture is defined as the values, attitudes, and behaviors that characterize a school.^27^ School culture has been shown to influence student substance use.^27^ Thus, it stands to reason that school culture could similarly influence the stigma related to mental health. Our school staff interviews found an overall positive experience with Aevidum programming and a sense among staff that students were more open to discussing mental health. These findings were echoed with InSciEdOut, which included teacher narratives to supplement student survey results.^26^ Similar to our study, teachers felt positive about the programming, and felt students were more open in discussing mental illness with greater acceptance of mental health diagnoses.^26^

Study strengths include post-pandemic timing with a large, diverse student sample. Limitations include a lower than expected post-survey response rate due in part to difficulties with school e-mail servers. Although the Aevidum curriculum showed significant improvements in adolescent mental health knowledge and help-seeking, its impact on long-term clinical outcomes was not assessed. Schools had previously worked with the team or expressed interest in Aevidum, so results may not generalize to other educational settings without assessment of contextual, socioeconomic, and cultural differences. Also, students who participated may not represent the general adolescent population.

## Implications and Next Steps

This study contributes to the growing body of literature on school-based depression education and peer support programming. Future investigations should consider the long-term impact of the curriculum, especially sustained changes in knowledge and help-seeking with annual exposure (Aim 1). Response rates may be improved by including the assessment in the classroom at the end of curriculum delivery. This may also improve the ability to capture a broader spectrum of student opinions.

Aevidum club did not demonstrate significantly greater impact vs the curriculum alone in the short-term, but this may change long-term as clubs become more established and grow their membership (Aim 2). Finally, while staff noted positive changes, barriers to implementation were also identified. Implementation of evidence-based mental health practices in the school setting is an emerging area of research that could support sustaining Aevidum programming in the school setting (Aim 3).^28^

**SO WHAT?**

**What is already known on this topic?**

Research on school-based depression education and peer support programs demonstrates improved knowledge and help-seeking. Impact on school culture and reducing stigma is mixed.

**What does this article add?**

The Aevidum curriculum and club are in widespread use, but lack formal evaluation of impacts on knowledge, help-seeking, and culture change. This evaluation considered Aevidum curriculum and club components, and added school staff perspective to better understand school culture outcomes.

**What are the implications for health promotion practice or research?**

Students reported improved knowledge and help-seeking intention with Aevidum; school staff reported positive school culture changes not captured in student surveys. This suggests consistent, annual curriculum and club exposure may have more widespread impact in supporting adolescent mental health.
